# TrkB activation mitigates blast-induced cochlear pathology and promotes auditory recovery in a compressed-air blast model

**DOI:** 10.3389/fneur.2026.1822487

**Published:** 2026-05-28

**Authors:** Han-Gyu Bae, Sung Kyun Kim, Ashley Park, Alex Vandenberg, Jeoung Soo Lee, Jun Hee Kim

**Affiliations:** 1 Kresge Hearing Research Institute, Department of Otolaryngology-Head and Neck Surgery, University of Michigan, Ann Arbor, MI, United States; 2 Drug Design Development Delivery (4D) Laboratory, Department of Bioengineering, Clemson University, Clemson, SC, United States; 3 Department of Cell and Developmental Biology, University of Michigan, Ann Arbor, MI, United States

**Keywords:** 7,8-DHF, BDNF, blast, hearing loss, PgP nanoparticle

## Abstract

Blast-induced hearing loss (BIHL) is a prevalent form of sensory neurotrauma resulting from military and occupational blast exposure. However, effective post-injury interventions remain limited, and progress is hindered by challenges in delivering therapeutics to the injured cochlea. In this study, we developed a reproducible compressed-air blast mouse model with quantified output characteristics and evaluated a localized post-blast TrkB activation strategy using 7,8-dihydroxyflavone (7,8-DHF) delivered to the cochlea via poly(lactide-co-glycolide)-graft-polyethylenimine (PgP) nanoparticles. Device characterization demonstrated a monotonic relationship between regulator-setting pressure (100–250 psi) and acoustic output (72–124 dB). In addition, device modification increased acoustic output while reducing peak pressure relative to the prior configuration, thereby enabling reproducible, pressure-dependent injury. A single unilateral blast exposure produced robust elevations in auditory brainstem response (ABR) thresholds that partially recovered at intermediate regulator settings; however, these thresholds remained elevated over time at higher regulator settings. Since the modified blast paradigm frequently produced tympanic membrane perforation, we leveraged this transient access route to deliver 7,8-DHF-loaded nanoparticles through the ear canal immediately after blast. Compared with vehicle treatment, local 7,8-DHF delivery accelerated functional recovery and yielded significantly improved ABR thresholds, whereas vehicle-treated mice remained persistently impaired at 1 month. Histological analyses revealed preserved inner hair cell density, but significant outer hair cell loss and reduced inner hair cell ribbon synapses in mice exposed to blast. 7,8-DHF-loaded PgP attenuated outer hair cell loss and partially preserved ribbon synapses, with the strongest protection in basal cochlear regions. Together, these findings provide an accessible BIHL platform and support nanoparticle-enabled local TrkB activation as a feasible post-blast strategy that improves both functional and structural endpoints relevant to sensory neurotrauma.

## 1 Introduction

Blast exposure is a common cause of auditory dysfunction in both military personnel and civilians, often producing hearing deficits that are persistent and difficult to treat. Blast-induced hearing loss (BIHL) is particularly relevant to U.S. veterans: the U.S. Department of Veterans Affairs lists hearing loss among the most prevalent service-connected disabilities, affecting more than 4.7 million veterans in 2024 (1). Much of this burden is believed to arise from repeated exposure to high-intensity impulse noise, including weapons fire and explosive blasts (2). Consistent with this finding, studies of U.S. Army personnel deployed to Iraq and Afghanistan report that approximately 15–20% experienced symptoms consistent with BIHL (3, 4). Together, these observations underscore the need to define the mechanisms of BIHL and to develop effective strategies for prevention and treatment.

Progress toward mechanism-informed therapies has been slowed, in part, by limitations in available preclinical models. Existing rodent blast paradigms can differ markedly in waveform characteristics, peak overpressure, and injury severity, complicating cross-study comparisons and limiting therapeutic screening. Moreover, many commonly used platforms require costly infrastructure and specialized equipment (e.g., shock tubes, blast chambers, or explosive detonations), reducing accessibility and throughput (5). To overcome this barrier, a cost-efficient, paintball device-based blast mouse model was developed and used primarily to study mild traumatic brain injury (6, 7). With appropriate modification and optimization, this approach could provide an accessible platform for auditory blast research and for preclinical testing of candidate otoprotective therapies.

At the tissue level, blast exposure can produce both conductive and sensorineural components of hearing impairment. Conductive deficits commonly result from tympanic membrane perforation, whereas sensorineural dysfunction reflects injury to cochlear sensory and neural elements. While conductive hearing loss can often be managed surgically, effective treatments for the sensorineural component of BIHL remain limited (8). Blast exposure can damage multiple cochlear structures, including loss of outer hair cells (OHCs), ribbon synapse reduction at inner hair cells (IHC), and degeneration of spiral ganglion neurons (2, 8–10). These cellular phenotypes are strongly associated with auditory dysfunction and provide quantifiable endpoints for evaluating candidate interventions. However, therapeutic strategies that preserve cochlear structure and accelerate functional recovery after blast remain an unmet need.

One promising pathway is BDNF/TrkB signaling, which supports neuronal survival, synaptic maintenance, and activity-dependent plasticity. Pharmacologic activation of TrkB using 7,8-dihydroxyflavone (7,8-DHF) has shown protective or restorative effects in models of noise-induced sensorineural hearing loss (11–13). Importantly, noise- and blast-induced hearing loss share key phenotypic features, including threshold elevation in the auditory brainstem responses and synaptopathic changes, suggesting that TrkB activation may also be beneficial in BIHL. Translating this concept, however, requires overcoming cochlear delivery barriers and enabling rapid post-injury intervention. Notably, tympanic membrane perforation after blast can provide a temporary route for local, timely drug delivery directly through the ear canal. In addition, nanoparticle-based formulations can improve local retention and delivery of small molecules such as 7,8-DHF, a TrkB agonist. In particular, poly(lactide-co-glycolide) (PLGA) polymers have been evaluated as biodegradable drug-delivery platforms because of their favorable safety profile, biocompatibility, and ability to provide sustained release of therapeutic agents (14). Poly-lactic-co-glycolic acid (PLGA) nanoparticles undergo hydrolytic degradation into lactic acid and glycolic acid, which are subsequently metabolized through endogenous pathways. Drug release from these nanoparticles occurs through a combination of diffusion from the polymer matrix and gradual polymer degradation, allowing prolonged local exposure. Biodegradable PLGA nanoparticles have been demonstrated to escape endolysosomal components into the cytoplasm and release encapsulated compounds intracellularly over an extended period (15). These characteristics make PLGA nanoparticles highly suitable for delivering therapeutic compounds to tissues that are difficult to access and to small target structures, such as the cochlea, where local drug retention and controlled release are critical.

In the present study, we adapted and modified a paintball device-based blast paradigm to establish a reproducible mouse model of BIHL and to define pressure-dependent auditory outcomes. We then tested whether TrkB activation via 7,8-DHF formulated in poly(lactide-co-glycolide)-graft-polyethylenimine (PgP) nanoparticles (6) can improve recovery after blast exposure using longitudinal ABR measurements and cochlear histological structure (hair cell survival and IHC ribbon synapses) to demonstrate functional and structural recovery. By combining a scalable, well-characterized blast platform with a targeted, locally deliverable TrkB agonist therapy, this study establishes a practical framework for mechanistic studies and therapeutic screening in blast-related auditory injury.

## 2 Methods

### 2.1 Animals

Eight-week-old C57BL/6 J mice were used in this study. All mice were housed in a 12-h light/dark cycle with *ad libitum* access to food and water. All experimental procedures were approved by the Institutional Animal Care and Use Committee (IACUC) of the University of Michigan (Approval No.: PRO00011243) and were conducted in accordance with the National Institutes of Health guidelines for the care and use of laboratory animals.

### 2.2 Measurement of output pressure and sound intensity of the blast system

A low-cost blast system was built using a paintball device (Mini GS, Empire Paintball, Sewell, NJ, United States) connected to a compressed air tank (48/3000 HPA tank, Maddog Sports, CA, United States) and securely mounted in a fixed position, following a previously published study (16). To optimize the system for auditory studies, we modified the outlet of the device from 6 mm to 25 mm.

To characterize the blast output, measurements of both output pressure and sound intensity were performed across a range of regulator settings of the device (100 psi to 250 psi). Output pressure was measured using a pressure transducer (Honeywell, Morristown, NJ, United States) positioned at the location where the targeted region would be placed during experiments (1 cm away from the device outlet). The pressure transducer was set to record pressure readings every 20 ms to capture the dynamic changes in pressure during the blast. The voltage reading from the pressure transducer was back-calculated to psi using an Arduino chip board. By reading the baseline as 14.5038 psi of atmospheric pressure, additional pressure was calculated by subtracting the reading values from the baseline. To reduce measurement variability, pressure outputs at each regulator setting were recorded from 20 independent blasts.

Sound intensity measurements were conducted using a decibel meter (HT-80A, RISEPRO, China) positioned at the location where the target region would be placed during experiments (1 cm from the device outlet). For accurate measurement of sound intensity, 10 independent recordings were conducted for each regulator setting. In each recording, maximum sound intensity was captured from 5 to 10 blasts.

### 2.3 Blast-induced hearing impairment

Mice were anesthetized with 3.0% isoflurane (induction) and maintained at 2.5% (1 L/min O2), and they were restrained in a 3D-printed mesh mouse holder mounted on a manipulator stage (Velmex Inc., Bloomfield, NY, United States). The mouse heads were located 1 cm from the outlet of the blast device, and high-pressure air was released through the 25 mm device outlet by manual triggering.

### 2.4 Auditory brainstem response

Auditory brainstem responses (ABRs) on the blast-exposed side of the ear were acquired from control (no blast exposure), vehicle (blast with empty nanoparticle treatment), and DHF-treated (blast with 7,8-DHF-loaded nanoparticle treatment) mice at different time points during the recovery period, both before and after blast injury (at 1 day, 1 week, and 4 weeks). ABRs were recorded to assess hearing thresholds. Mice were anesthetized with 3.0% isoflurane (induction) and maintained at 2.5% (1 L/min O2). ABR recordings were performed inside a sound-attenuating chamber (Med Associates, St. Albans, VT, United States) with subdermal electrodes (Rochester Electro-Medical, Lutz, FL, United States) placed at the vertex (active), ipsilateral mastoid (reference), and contralateral mastoid (ground). Acoustic stimuli were generated by an Auditory Evoked Potentials Workstation (Tucker-Davis Technologies, Alachua, FL, United States). Clicks (0.1 ms duration, amplitude-modulated square waves) were delivered to the left ear via a closed-field system using a 10-cm plastic tube (Tygon; 3.2 mm outer diameter) connected to TDT Multi-Field Magnetic Speakers. Click stimuli intensities ranged from 90 to 10 dB sound pressure level (SPL) (5 dB decrements), with 512 recordings averaged (16 Hz sampling rate). Tone stimuli (4, 8, 12, 16, and 32 kHz) were presented at intensities ranging from 90 to 20 dB SPL (10 dB decrements), with the same sampling rate.

### 2.5 Preparation of 7,8-dihydroxyflavone-loaded PgP nanoparticle

PgP was synthesized as described in the previous publication (6). 7,8-Dihydroxyflavone (7,8-DHF, Sigma, St. Louis, MO, United States) loading into PgP was achieved by solvent evaporation (6). 7,8-DHF was dissolved in ethanol (1 mg DHF/mL ethanol), and 500 μL of 7,8-DHF was added to 1 mL PgP (1 mg PgP /mL water). The mixture was incubated for 4 h at room temperature in the dark, followed by evaporation of ethanol overnight. The 7,8-DHF-loaded PgP nanoparticles were filtered using a cellulose acetate membrane syringe filter (molecular weight cut-off [MWCO]): (0.2 μm, cellulose acetate, Nalgene, Thermo Scientific, United States) to remove any precipitated 7,8-DHF. The amount of 7,8-DHF-loaded PgP nanoparticle was measured using a tandem LC–MS/MS system with an electrospray ionization (ESI) MS/MS detector interfaced with a capillary liquid chromatography (cap-LC, Waters Xevo TQ-S micro mass spectrometry system) (17). The Ultra-Performance Liquid Chromatography (UPLC) system was composed of a Waters Acquity Premier binary solvent manager and autoinjector. LC was performed using an XBridge™ Premier BEH Phenyl Column (2.1 mm × 150 mm, 3 μm, Waters, Milford, MA, United States). The mobile phase consisted of water + 0.05% trifluoroacetic acid (A) and acetonitrile (B). The flow rate was set at 0.4 mL/min with a gradient (80% A for 2 min, decreased to 10% A within 5 min, maintained 10% A for 1 min, and then increased back to 80% A within 1 min), and the total run time was 9 min. The effluent was directed to an electrospray ionization source system, and multiple reaction monitoring was used for acquisition. Retention time of 7,8-DHF was determined as approximately 4.25 min. The loading efficiency of 7,8-DHF-loaded into the PgP micelle core was calculated by the following equation:

$$\%\text{loading efficiency}=\frac{\text{Amount of}\;\mathrm{DHF}\;\text{loaded}}{\text{Aount of}\;\mathrm{DHF}\;\text{added}}\times 100$$

The % loading efficiency was 64% ± 8.22 (*n* = 3 replicates), and the concentration of 7,8-DHF (1 mg PgP in water) was 320.26 ± 41.1 μg/mL.

### 2.6 Intratympanic PgP nanoparticle injection

Both empty PgP (vehicle) and 7,8-DHF-loaded PgP were delivered immediately after blast exposure (within 1 h). The mice were continuously maintained under anesthesia from blast exposure and moved under stereomicroscopy to examine tympanic membrane perforation. With perforation of the tympanic membrane on the blast-exposed side of the ear, 10 μL of nanoparticles was slowly delivered through the perforated tympanic membrane using a Hamilton syringe with a 30 G needle. After injection, the mice were left for 5 min for the nanoparticles to be absorbed and diffused.

### 2.7 Statistical analysis

Experimental data were processed and visualized using Prism software (GraphPad Software, San Diego, CA, United States). To test statistical significance between groups, a two-way ANOVA with multiple comparisons was conducted, and the *p*-values were adjusted with Bonferroni correction. A *p*-value of < 0.05 was considered statistically significant.

## 3 Results

### 3.1 Compressed-air blast delivered by a paintball device induces pressure-dependent and, at higher settings, persistent hearing impairment

**Figure 1:**
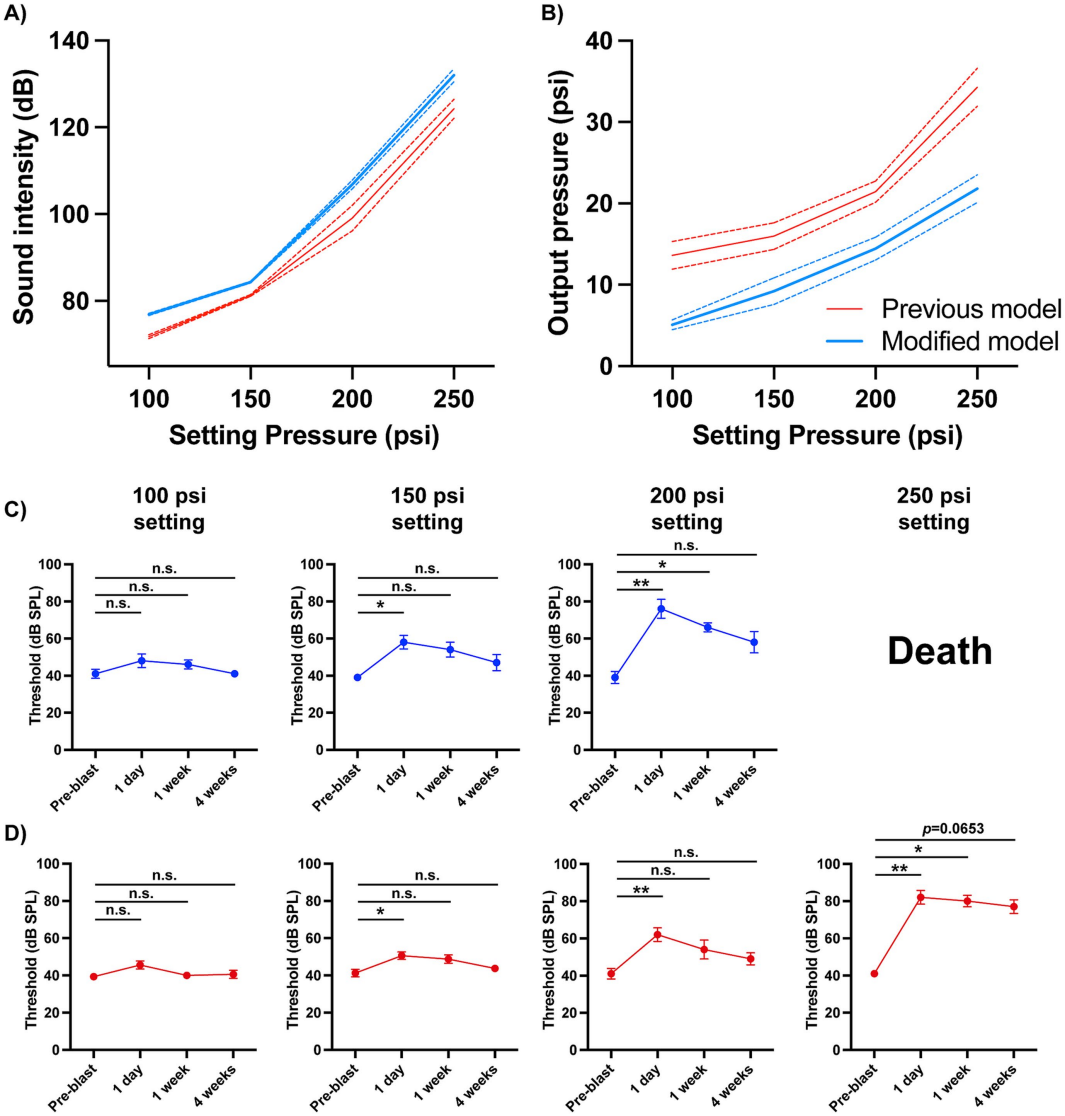
Calibration of the blast device and pressure-dependent auditory threshold shifts. **(A)** Peak sound intensity (dB) measured as a function of regulator setting pressure (psi) for the previous (red) and modified (blue) blast device. Solid lines indicate the mean across repeated measurements; dashed lines indicate variability across repeats. **(B)** Peak output pressure (psi) measured at the blast outlet as a function of regulator setting pressure, comparing the previous (red) and modified (blue) devices. **(C,D)** Hearing thresholds (dB SPL) of auditory brainstem responses (ABRs) measured pre-blast and at 1 day, 1 week, and 4 weeks post-blast for exposures delivered at the indicated setting pressures. **(C)** Threshold shifts following blasts generated with the previous original device (blue); the 250 psi setting resulted in mortality, preventing longitudinal threshold assessment (*n* = 5 for 100 psi, *n* = 5 for 150 psi, *n* = 5 for 200 psi). **(D)** Threshold shifts following blasts generated with the modified device (red), including the 250 psi condition (*n* = 8 for 100 psi, *n* = 8 for 150 psi, *n* = 5 for 200 psi, *n* = 5 for 250 psi). Data are shown as mean ± 95% CI **(A,B)** or s.e.m. **(C,D)**. The statistical significances were acquired using multiple comparisons from a one-way ANOVA test and represented with asterisks. * *p* < 0.05, ** *p* < 0.01; n.s., not significant.

Although a paintball device-based blast paradigm has been reported to elicit mild traumatic brain injury (16, 18), its effects on auditory function have not been systematically characterized. To optimize this paradigm for auditory injury studies, we compared a modified blast device with the previous design across regulator setting pressures ranging from 100 to 250 psi. Because the previous configuration produced relatively high overpressure for a given acoustic output, we modified the device by enlarging the output window of the paintball device (Supplementary Figure 1). In both configurations, peak sound intensity increased monotonically with regulator setting. In the previous device, sound output was gradually increased from 71.81 ± 0.664 dB (S.D.) to 124.31 ± 3.132 dB as the setting pressure increased from 100 to 250 psi. Across 100–250 psi, the modified paintball device generated higher sound intensity, ranging from 76.89 ± 0.40 dB at 100 psi to 132.03 ± 2.08 dB at 250 psi (Figure 1A). In the previous device, the output pressure increased from 13.61 ± 3.63 psi to 34.30 ± 5.05 psi as the regulator setting increased from 100 psi to 250 psi. In contrast, the modified paintball device generated lower output pressures, ranging from 5.07 ± 1.33 psi to 21.83 ± 3.69 psi at 100 psi and 250 psi, respectively. Notably, the modified device generated higher sound intensity at matched settings relative to the previous model, particularly at ≥200 psi. In contrast, peak output pressure measurements revealed that the modified device produced substantially lower output pressure than the previous design across the same regulator setting, indicating that the modification reduced overpressure while preserving acoustic output—features expected to better approximate key aspects of real-world blast exposure (Figure 1B).

To determine whether the paintball device generates a blast that produces hearing impairment, we delivered a single unilateral blast at the various regulator settings and longitudinally measured auditory brainstem response (ABR) thresholds from the exposed side of the ear (pre-blast, 1 day, 1 week, and 4 weeks post-blast). The regulator setting of 100 psi did not significantly alter ABR thresholds over time (41.00 ± 2.45 dB SPL pre-blast vs. 48.00 ± 3.74 dB SPL at 1 day; *p* = 0.2247; a one-way ANOVA with Bonferroni multiple comparisons; Figure 1C). Increasing the setting to 150 psi produced a significant acute threshold elevation at 1 day (39.00 ± 1.87 to 58.00 ± 3.74 dB SPL; *p* = 0.0057), followed by partial recovery at later time points. The setting of 200 psi induced a robust acute threshold shift at 1 day (39.00 ± 3.31 to 76.00 ± 5.10 dB SPL; *p* < 0.0001) that remained elevated at later time points, consistent with incomplete recovery by 4 weeks (Figure 1C). At the setting of 250 psi, animals did not survive, defining an upper boundary for usable exposure levels with the previous configuration.

With the modified device, ABR threshold shifts showed a similar pressure-dependent pattern (Figure 1D). While blast at the setting of 100 psi did not significantly increase thresholds (39.38 ± 1.48 to 48.63 ± 2.20 dB SPL; *p* = 0.0843), those were increased significantly when the mice exposed to the blast at the setting of 150 psi (41.25 ± 2.06 to 50.63 ± 1.99 dB SPL; *p* = 0.0091) and 200 psi (41.00 ± 2.92 to 62.00 ± 3.74 dB SPL; *p* = 0.0042). The setting of 250 psi produced a large acute elevation (41.00 ± 1.87 to 82.00 ± 3.74 dB SPL; *p* < 0.0001) and, importantly, thresholds remained significantly elevated after 4 weeks from blast exposure (77.00 ± 3.74 dB SPL; *p* < 0.0001) without any lethal consequence. In contrast, thresholds largely recovered toward baseline by 4 weeks following blast exposure at settings of 150 psi (43.75 ± 1.83 dB SPL; *p* > 0.9999) and 200 psi (49.00 ± 3.32 dB SPL; *p* = 0.4855; Figure 1D). Collectively, these data show that the modified device increases acoustic intensity while lowering output pressure and produces a controllable, pressure-dependent threshold shift relative to the previous design. This configuration enables reproducible induction of persistent hearing impairment at higher regulator settings (250 psi), with milder and largely reversible threshold elevations at intermediate regulator settings (150–200 psi).

### 3.2 Direct activation of BDNF/TrkB signaling promotes recovery from blast-induced hearing impairment

The BDNF/TrkB signaling pathway has been proposed to play a protective role in noise-induced hearing impairment (7, 19). Given the overlapping phenotypes observed in noise- and blast-induced hearing impairment (7, 20), we hypothesized that BDNF/TrkB signaling plays a protective role in blast-induced hearing impairment. We, therefore, tested whether pharmacological activation of the signaling pathway with the TrkB agonist, 7,8-DHF, loaded in PgP nanoparticles, enhances functional recovery after blast exposure. Notably, blasts generated with the modified configuration produced tympanic membrane perforation, enabling direct local delivery of 7,8-DHF PgP through the ear canal. To evaluate whether this rapid, local administration improves recovery from blast-induced hearing impairment, we measured ABR thresholds longitudinally (pre-blast and at 1, 7, and 28 days post-blast).

**Figure 2:**
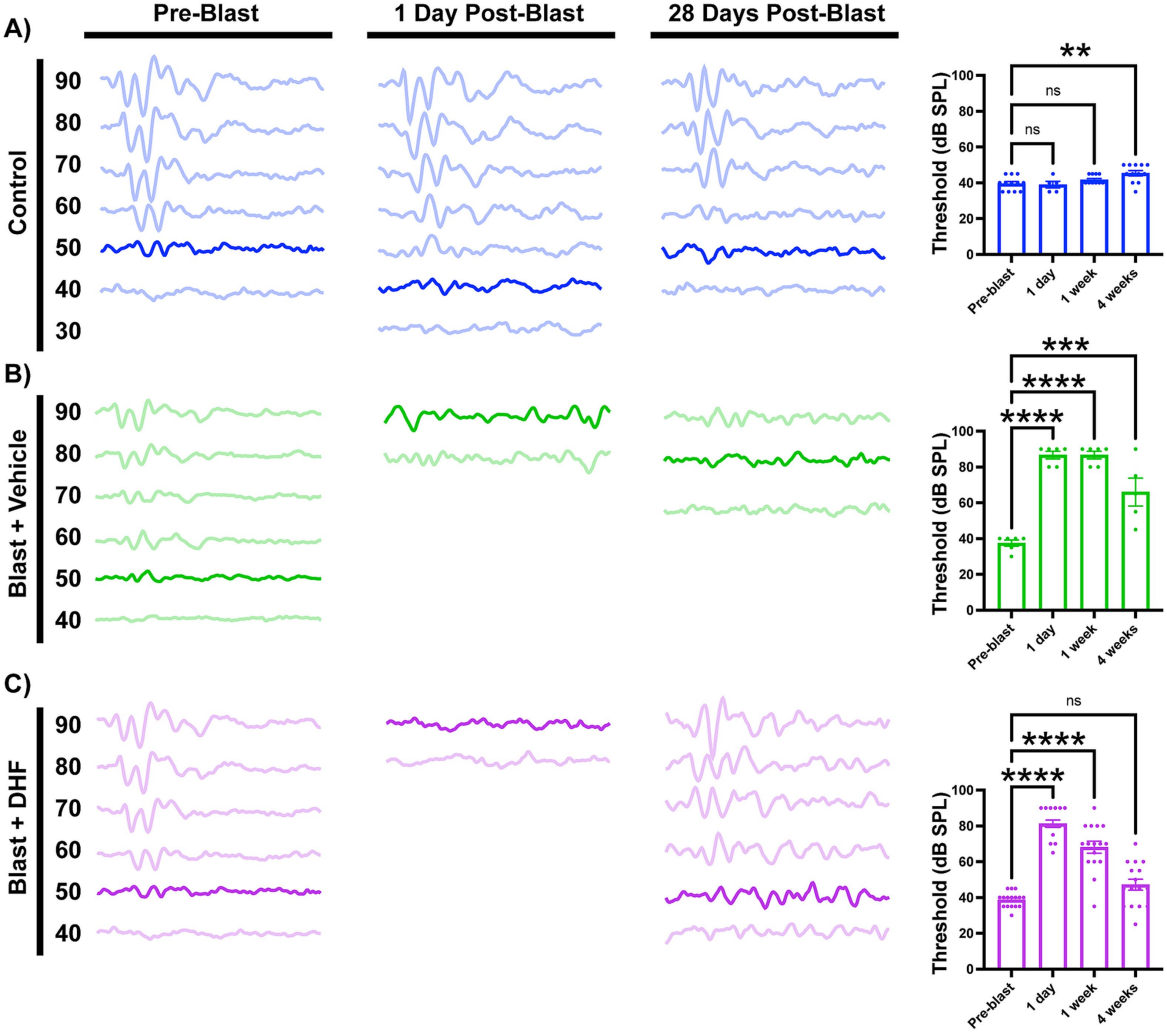
7,8-DHF-loaded PgP nanoparticles enhance recovery of ABR thresholds after blast exposure. **(A–C)** Representative ABR waveforms evoked by decreasing sound levels (dB SPL, left) recorded pre-blast, 1-day post-blast, and 28 days post-blast from **(A)** control (*n* = 12 mice), **(B)** blast + vehicle (*n* = 6 mice), and **(C)** blast + DHF treatment groups (*n* = 16 mice). Bar graphs (right) summarize ABR thresholds measured pre-blast and at 1, 7, and 28 days after blast. ABR threshold was defined as the lowest stimulus level that elicited a repeatable waveform. Data are presented as mean ± error (as plotted). Statistical significance was assessed within each group using a one-way ANOVA followed by Bonferroni multiple comparisons testing. Asterisks indicate post-hoc significance (** *p* < 0.01; *** *p* < 0.001; **** *p* < 0.0001); n.s., not significant.

In the control group, ABR thresholds remained relatively stable across sessions, with only modest changes observed over time, and waveform morphology was preserved at comparable stimulus levels before and after the experimental timeline (Figure 2A). In contrast, mice exposed to blast and treated with vehicle (empty PgP nanoparticle, thereafter Blast + Vehicle) exhibited a pronounced elevation in ABR thresholds at 1-day post-blast that persisted at 7 days, followed by partial recovery by 28 days; however, thresholds remained elevated relative to baseline (Figure 2B). ABR threshold in the Blast + Vehicle group increased from 37.50 ± 1.71 dB SPL to 86.67 ± 2.11 dB SPL at 1 day after blast exposure (*p* < 0.0001, multiple comparison from ANOVA with Bonferroni correction) and remained high after 1 month of recovery (66.00 ± 7.81 dB SPL, *p* < 0.0002, multiple comparison from ANOVA with Bonferroni correction).

Mice treated with 7,8-DHF-loaded PgP nanoparticles (hereafter referred to as Blast + DHF) showed a similar acute threshold elevation at 1 day after blast exposure, from 38.75 ± 1.07 dB SPL to 81.25 ± 2.07 dB SPL (*p* < 0.0001), but demonstrated a greater recovery trajectory over time (Figure 2C). By 4 weeks, thresholds were markedly lower (47.19 ± 3.06 dB SPL) and no longer significantly different from baseline (*p* = 0.0679), accompanied by re-emergence of clear ABR waveforms at lower stimulus intensities.

Together, these results indicate that local delivery of 7,8-DHF via PgP nanoparticles enhances functional recovery of auditory sensitivity after blast-induced hearing impairment, consistent with a protective role for BDNF/TrkB signaling pathway activation in this model.

### 3.3 Activation of BDNF/TrkB signaling prevents outer hair cell loss without affecting inner hair cell number

**Figure 3:**
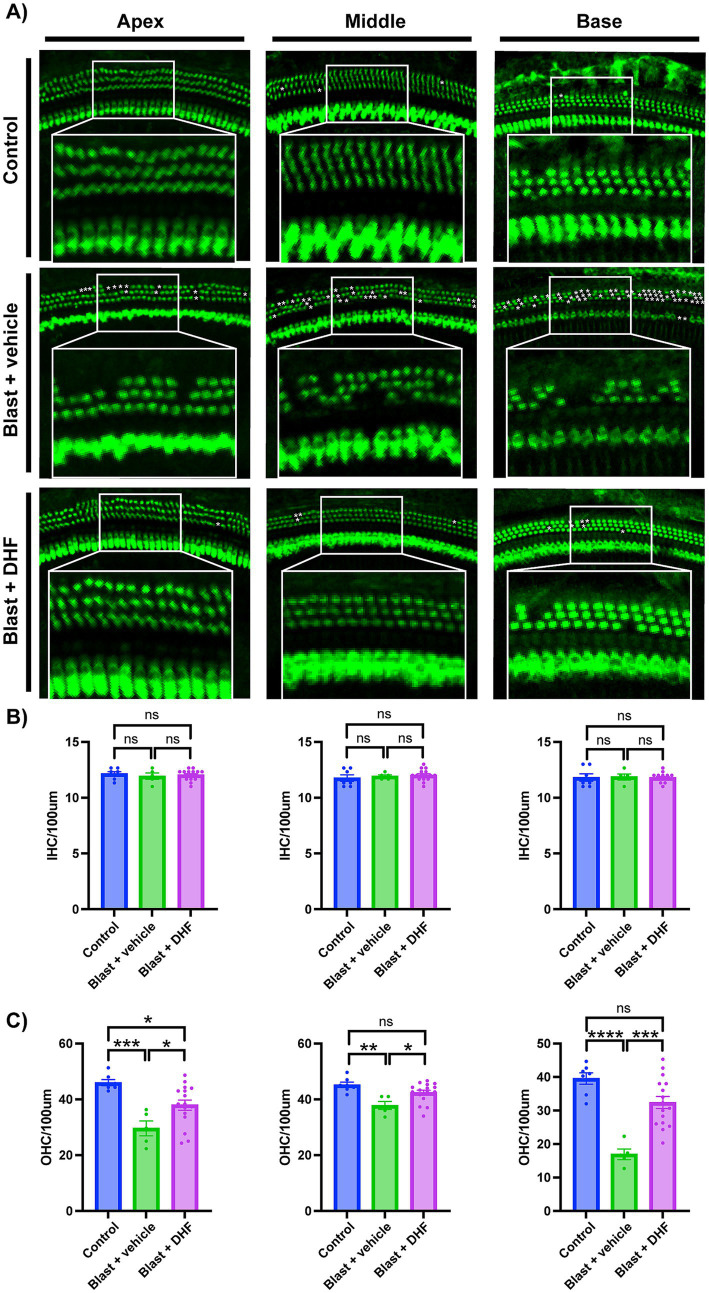
Blast exposure preferentially reduces outer hair cell survival, and 7,8-DHF-loaded PgP nanoparticle treatment attenuates outer hair cell loss. **(A)** Representative cochlear mount images immunolabeled for Myosin VIIa (Myo7a) to visualize sensory hair cells in apical, middle, and basal regions. Images are shown for control (no blast, *n* = 7 mice), blast + vehicle (*n* = 5 mice), and blast + DHF groups (*n* = 16 mice, top to bottom). Boxed regions indicate areas enlarged below each low-magnification image. **(B,C)** Quantification of inner hair cell (IHC) density (B; IHC/100 μm) and outer hair cell (OHC) density (C; OHC/200 μm) in apical, middle, and basal regions for each group. Data are presented as mean ± error (as plotted). Statistical significance was determined using a one-way ANOVA with Bonferroni multiple comparisons testing and is denoted by asterisks (* *p* < 0.05; ** *p* < 0.01; *** *p* < 0.001; n.s., not significant).

To determine whether blast exposure induces cochlear sensory cell loss and whether TrkB activation confers protection, we assessed hair cell integrity using Myo7a immunolabeling in cochlear mounts and quantified IHC and OHC densities along the cochlear axis (apex, middle, and base; Figure 3). Across all regions, IHC density was preserved, with no significant loss among the Control, Blast + Vehicle, and Blast + DHF groups (Figures 3A,B). The result of comparable IHC densities between blast-exposed mice (apex, 11.93 ± 0.29; middle, 11.93 ± 0.12; base, 11.87 ± 0.27 cells/100 μm) and controls (apex, 12.14 ± 0.19; middle, 11.76 ± 0.29; base, 11.81 ± 0.33 cells/100 μm; all *p* > 0.9999) suggests that blast exposure in the modified model does not induce any IHC loss.

In contrast, blast exposure produced a marked reduction in OHC density, indicating preferential OHC vulnerability (Figures 3A,C). OHC loss was evident in the Blast + Vehicle group across apical, middle, and basal regions, with significant decreases relative to controls. In the Blast + Vehicle group, OHC densities decreased to 29.60 ± 2.69, 37.80 ± 1.43, and 16.93 ± 1.58 cells/100 μm in the apical, middle, and basal regions, respectively, compared with 46.00 ± 1.06, 45.19 ± 0.96, and 39.57 ± 1.72 cells/100 μm in control group (apex, *p* = 0.0005; middle, *p* = 0.0023; base, *p* < 0.0001).

Treatment with 7,8-DHF in the Blast + DHF groups mitigated blast-associated OHC loss in a region-dependent manner. In the middle and basal cochlea, OHC densities increased to 42.38 ± 0.89 and 32.40 ± 1.80 cells/100 μm, respectively, and were not significantly different from control values (middle, *p* = 0.2134; base, *p* = 0.0506), consistent with substantial structural preservation, particularly in the basal region that showed the greatest vulnerability after blast. In the apex, OHC density showed only a modest improvement (37.96 ± 1.84 cells/100 μm) and remained reduced relative to the control group, without a significant difference compared with Blast + Vehicle group (Figure 3C).

Together, these results indicate that blast exposure primarily compromises outer hair cell survival while sparing inner hair cells and that local TrkB activation via 7,8-DHF-loaded PgP provides measurable protection, most prominently in the basal cochlea.

### 3.4 Activation of BDNF/TrkB signaling partially preserves IHC ribbon synapses after blast exposure

**Figure 4:**
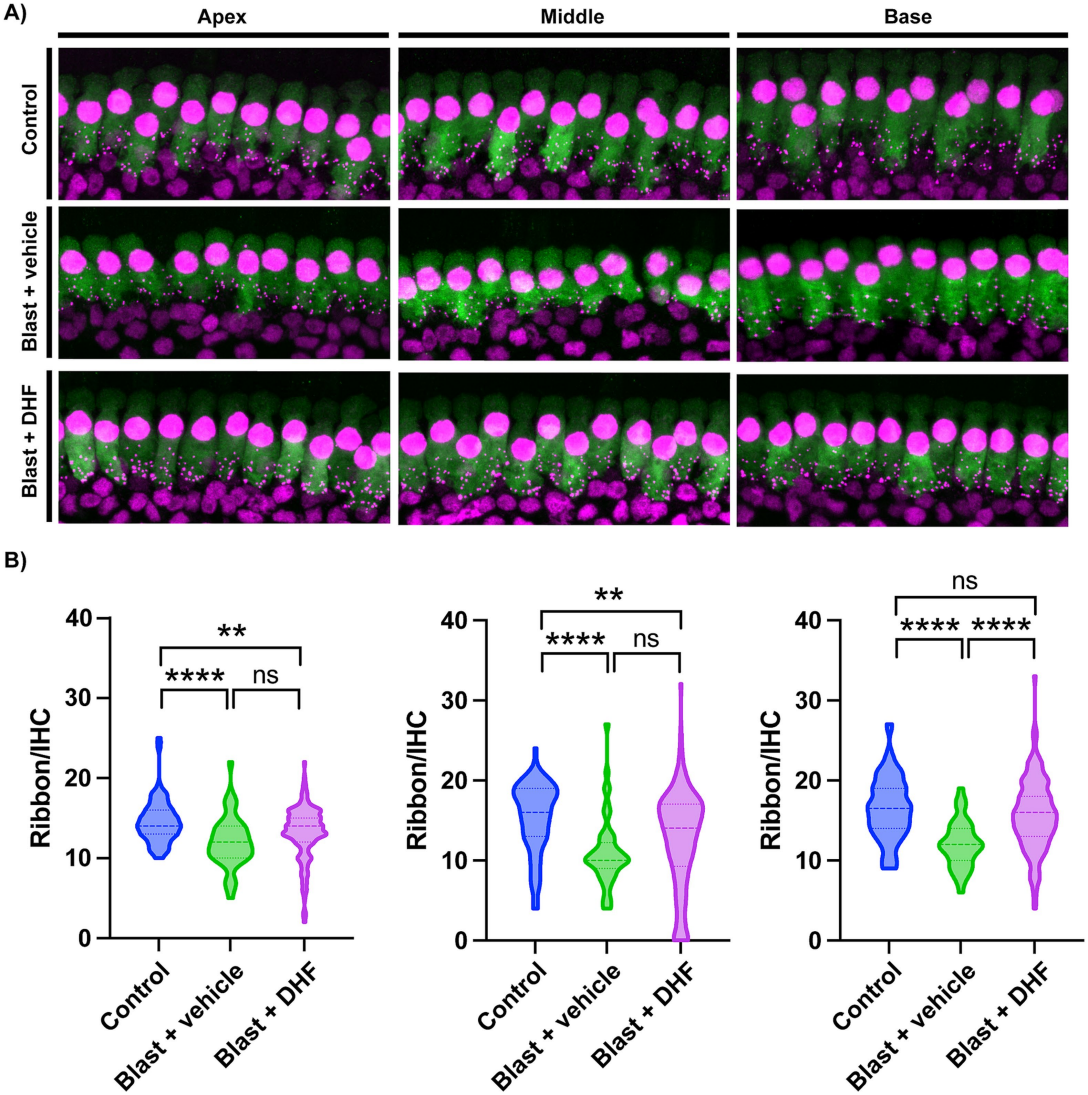
Blast exposure reduces IHC ribbon synapse number, and 7,8-DHF–PgP treatment preserves ribbon synapses in a region-dependent manner. **(A)** Representative confocal images of cochlear whole mounts from the Control, Blast + Vehicle, and Blast + DHF groups. Inner hair cells (IHCs) were immunolabeled with Myo7a (magenta), and presynaptic ribbons were labeled with CtBP2 (green). Images are shown from the apex, middle, and base (left to right). **(B)** Quantification of ribbon synapses per IHC (Ribbon/IHC) in apical (*n* = 70 cells from 7 Control mice, *n* = 50 cells from 5 Blast + Vehicle mice, and *n* = 160 cells from 16 Blast + DHF mice), middle (*n* = 70 cells from 7 Control mice, *n* = 50 cells from 5 Blast + Vehicle mice, and *n* = 160 cells from 16 Blast + DHF mice), and basal cochlear regions for each group (*n* = 70 cells from 7 Control mice, *n* = 49 cells from 5 Blast + Vehicle mice, and *n* = 160 cells from 16 Blast + DHF mice). Data are displayed as distribution plots with summary statistics (median values, bold dashed line; interquartile values, thin dashed line). Statistical significance was assessed using one-way ANOVA followed by Bonferroni multiple-comparisons testing. ** *p* < 0.01; **** *p* < 0.0001.

Because blast exposure can cause cochlear synaptopathy even when sensory cell loss is limited, we next assessed whether blast injury disrupts IHC ribbon synapses and whether TrkB activation mitigates this effect. Cochlear whole mounts were immunolabeled for Myo7a (IHCs) and CtBP2 (presynaptic ribbons), and ribbons were quantified as ribbons per IHC in the apical, middle, and basal regions (Figure 4).

In the Blast + Vehicle group, CtBP2 puncta were visibly reduced relative to the control group across cochlear regions (Figure 4A), suggesting a loss of ribbon synapses. Quantification confirmed a significant reduction in Ribbon/IHC following blast exposure, with a majority of robust differences evident in the middle and basal cochlea (Figure 4B). Specifically, ribbon numbers decreased from 14.56 ± 0.33, 15.37 ± 0.52, and 16.49 ± 0.47 ribbons/IHC in the apical, middle, and basal regions, respectively, in the control group, to 12.02 ± 0.45, 10.98 ± 0.60, and 12.04 ± 0.41 ribbons/IHC after blast exposure (apex, *p* < 0.0001; middle, *p* < 0.0001; base, *p* < 0.0001; multiple comparison from ANOVA with Bonferroni correction). Treatment with 7,8-DHF attenuated blast-induced ribbon synapse loss (Figures 4A,B). The protective effect was region-dependent, with the strongest preservation observed in the basal cochlea, where Ribbon/IHC values in the Blast + DHF group were comparable to those in the No-blast control group and were significantly higher than those in the Blast + Vehicle group (Figure 4B). In the apex and middle regions, 7,8-DHF produced a partial improvement in Ribbon/IHC relative to the Blast + Vehicle group (apex, 13.23 ± 0.25; middle, 12.76 ± 0.48 ribbons/inner hair cell), although this did not reach statistical significance (Figure 4B).

Together, these findings indicate that blast exposure induces IHC synaptopathy characterized by reduced ribbon synapse number and that local delivery of 7,8-DHF via PgP nanoparticles preserves ribbon synapses, most prominently in the basal cochlea.

## 4 Discussion

In this study, we established and validated a practical blast-induced hearing loss model using a paintball device-based compressed-air platform and used it to test a targeted post-blast therapeutic strategy. The modified device defined the relationship between regulator setting and acoustic/pressure outputs, enabling reproducible selection of blast conditions that produce graded auditory injury. Unilateral blast exposure induced robust, pressure-dependent ABR threshold elevations with partial recovery over time at intermediate settings and persistent deficits at higher injury levels. Blast injury preferentially affected cochlear structures associated with sensorineural hearing loss, most notably OHC survival and IHC ribbon synapses, with sparing of IHC density. Local TrkB activation using 7,8-DHF-loaded PgP nanoparticles enhanced functional recovery and provided measurable structural protection, particularly in vulnerable cochlear regions.

### 4.1 Optimization and reproducibility of a modified cost-efficient blast platform

A persistent challenge in blast auditory research is that preclinical models vary widely in blast waveform characteristics, peak overpressure, and injury severity, limiting cross-study comparison and complicating therapeutic screening. In addition, high cost and heavy equipment lower accessibility to study BIHL. However, recent research developed a small and cost-friendly blast-induced traumatic brain injury mouse model (16), which can be applicable for hearing research. In this study, by directly measuring sound intensity and peak output pressure across regulator settings and comparing device configurations, we provide a straightforward framework for selecting blast parameters that are both survivable and effective for generating consistent auditory outcomes. To mimic more of a traditional blast model and optimize it for the hearing study, we modified the blast device by enlarging the output. Compared to the previous blast device, the modified device can generate lower pressure output and louder sound intensity. Similar to the previous blast device, the modified blast device can induce temporary hearing impairment with a low-pressure setting. However, with the highest pressure setting, it can induce permanent hearing impairment in the mice. We have observed that the blast perforates the tympanic membrane, causing conductive hearing impairment. Given that the perforated tympanic membrane is recovered and closed within 7–10 days (21), the persistence of hearing impairment after 1 month of recovery suggests that the blast from the modified model induces sensory hearing impairment as well. These demonstrates the efficacy of modified blast model by mimicking a real blast. This research is necessary for laboratories seeking a scalable methods for blast research without access to shock tubes or blast chambers.

### 4.2 Blast exposure produces persistent auditory dysfunction linked to OHC loss and ribbon synapses

Blast exposure can produce both conductive and sensorineural hearing impairment. In our paradigm, tympanic membrane perforation occurred (particularly with the modified configuration), which likely contributes to early post-blast threshold elevation through a conductive component. However, the persistence of elevated ABR thresholds at later time points with cochlear injury supports a substantial sensorineural contribution that cannot be explained solely by transient middle-ear pathology. The observed time course showing a marked threshold elevation at 1 day, followed by incomplete recovery, is consistent with clinical and experimental observations that blast can initiate both acute mechanical disruption and longer-lasting cochlear degeneration and synaptic injury (2, 22, 23).

Our histological analyses revealed that blast exposure preferentially compromised OHC survival, while IHC density remained stable across cochlear regions. This pattern is consistent with the established vulnerability of OHCs to intense acoustic/impulsive injury and supports OHC loss as one structural correlate of persistent threshold elevation in this model. In parallel, we observed significant reductions in IHC ribbon synapses (CtBP2 puncta per IHC) across tonotopic locations, indicating blast-induced cochlear synaptopathy. Compared with previous research on blast-induced hearing loss, where basal outer hair cell loss was observed (2), the pathological conditions induced by our modified blast gun are more severe than those induced by the traditional blast tube. Because synaptic loss can occur even when hair cell survival is largely preserved, ribbon synapse quantification provides an additional mechanistic endpoint that may better capture functional deficits and recovery potential beyond threshold alone. The regional pattern of injury and protection—most prominent in middle-to-basal regions—aligns with known tonotopic gradients of susceptibility and is consistent with the functional severity observed in ABR outcomes.

### 4.3 BDNF/TrkB signaling is a candidate pathway to treat blast-induced hearing impairment

The BDNF/TrkB signaling pathway has been suggested as a candidate target for various hearing impairments, including noise-induced hearing impairment. Blast-induced hearing impairment and noise-induced hearing impairment share several cochlear pathologies, including outer hair cell damage, loss of inner hair cell ribbon synapses, auditory nerve dysfunction, oxidative stress, and inflammatory responses (7, 20, 24, 25). These overlapping features suggest that common molecular mechanisms, including BDNF/TrkB signaling, may contribute to cochlear injury and recovery after acoustic trauma. Therefore, it is hypothesized that modulation of signaling pathways known to be protective in noise-induced hearing impairment may also be a potential therapeutic strategy for blast-induced hearing impairment. Indeed, the BDNF/TrkB signaling pathway has been demonstrated to play a protective role in noise-induced hearing impairment (7). In the research, the research team modulated BDNF/TrkB signaling by systemically treating BDNF-enriched mesenchymal stem cell-derived small extracellular vesicles to deliver them into the cochlea through the blood–labyrinth barrier. The treatment significantly attenuated noise-induced hearing impairment as well as hair cell loss and ribbon synapse reduction (7). However, mesenchymal stem cell-derived small extracellular vesicles may contain multiple types of proteins and RNAs, which can lead to off-target modulation of signaling pathways (26). Therefore, we tested the 7,8-DHF-loaded PgP nanoparticle, which specifically modulates the BDNF/TrkB signaling pathway. In addition, blast-induced hearing impairment often accompanies tympanic membrane perforation, which provides an advantage for local cochlear treatment compared with noise-induced hearing impairment. Notably, we demonstrated that local treatment of 7,8-DHF to the cochlea after blast exposure enhances ABR threshold recovery during the recovery period. This recovery is accompanied by a reduction in outer hair cell loss and afferent synaptic loss in inner hair cells. In turn, we confirm that activation of the BDNF/TrkB signaling pathway plays a protective role in blast-induced hearing impairment as well. The role of BDNF/TrkB has been largely documented in the brain, including neuronal survival, structural changes, and plasticity (27). Among these, the possible protective molecular mechanisms in the cochlear nerve might include the amelioration of oxidative stress (28), as previous studies have also demonstrated (7). Given the distribution of TrkB in the neuronal population, specifically spiral ganglion neurons and neuronal terminals underneath the outer hair cells (29), the activation of those neurons may protect against outer hair cell loss and afferent synaptic loss from blast. Future studies assessing cochlear TrkB pathway activation (e.g., phosphorylated TrkB and downstream signaling) and evaluating synaptic function (e.g., ABR wave I amplitude and latency) will help clarify whether structural preservation translates to improved neural encoding beyond threshold recovery. However, blast injury also has distinctive features from other types of hearing impairment. Unlike conventional noise-induced hearing impairment, blast exposure generates a rapid pressure wave that can damage multiple auditory structures, including the tympanic membrane, middle ear, cochlea, auditory nerve, and central auditory pathways. Therefore, blast-induced hearing impairment may involve both conductive and sensorineural components. These differences should be considered when interpreting therapeutic effects, and future studies will be needed to distinguish the cochlear, middle-ear, and central contributions to TrkB-mediated protection.

### 4.4 Local delivery enabled by tympanic membrane perforation and nanoparticle formulation

A key translational barrier in cochlear therapeutics is drug delivery, particularly when rapid intervention is required following injury. In our model, blast-associated tympanic membrane perforation created a temporary route for direct local administration via the ear canal, enabling timely post-blast delivery without systemic dosing. Incorporating 7,8-DHF into PgP nanoparticles provides additional advantages, including improved local retention and controlled exposure compared with the free drug. Together, these features make the therapeutic approach used in this study conceptually aligned with real-world needs in blast settings, where rapid local interventions may be feasible. Nevertheless, further study is needed to quantify inner ear drug exposure, evaluate safety with repeated dosing, and define the therapeutic window for maximal benefit.

In addition, several limitations should be considered. First, our functional assessment focused on ABR thresholds. Suprathreshold neural deficits, temporal processing impairments, or central auditory consequences may persist even when thresholds recover. Second, we tested a single dosing strategy and time window. In the future, defining dose–response relationships, delayed-treatment efficacy, and the durability of structural protection using various methods, such as fluorescent-labeled nanoparticles and post-treatment assessment of TrkB activity, will be important for translational relevance.

In summary, this study establishes an optimized, accessible blast-hearing-loss platform that produces reproducible auditory dysfunction with quantifiable cochlear pathology. Using this model, we demonstrate that local TrkB activation via 7,8-DHF-loaded PgP nanoparticles improves functional recovery and partially preserves cochlear structure after blast exposure. These findings support TrkB signaling as a mechanistically motivated therapeutic target for BIHL and provide a practical preclinical framework for screening otoprotective interventions under controlled blast conditions.

## Data Availability

The original contributions presented in the study are included in the article/Supplementary material, further inquiries can be directed to the corresponding author.
