# Supplementary material for: TrkB activation mitigates blast-induced cochlear pathology and promotes auditory recovery in a compressed-air blast model
Source: Front Neurol. 2026 May 28;17:1822487. doi: 10.3389/fneur.2026.1822487 (PMC13253399; doi:10.3389/fneur.2026.1822487)
Supplement: Supplementary file 1 [file Image_1.PDF]

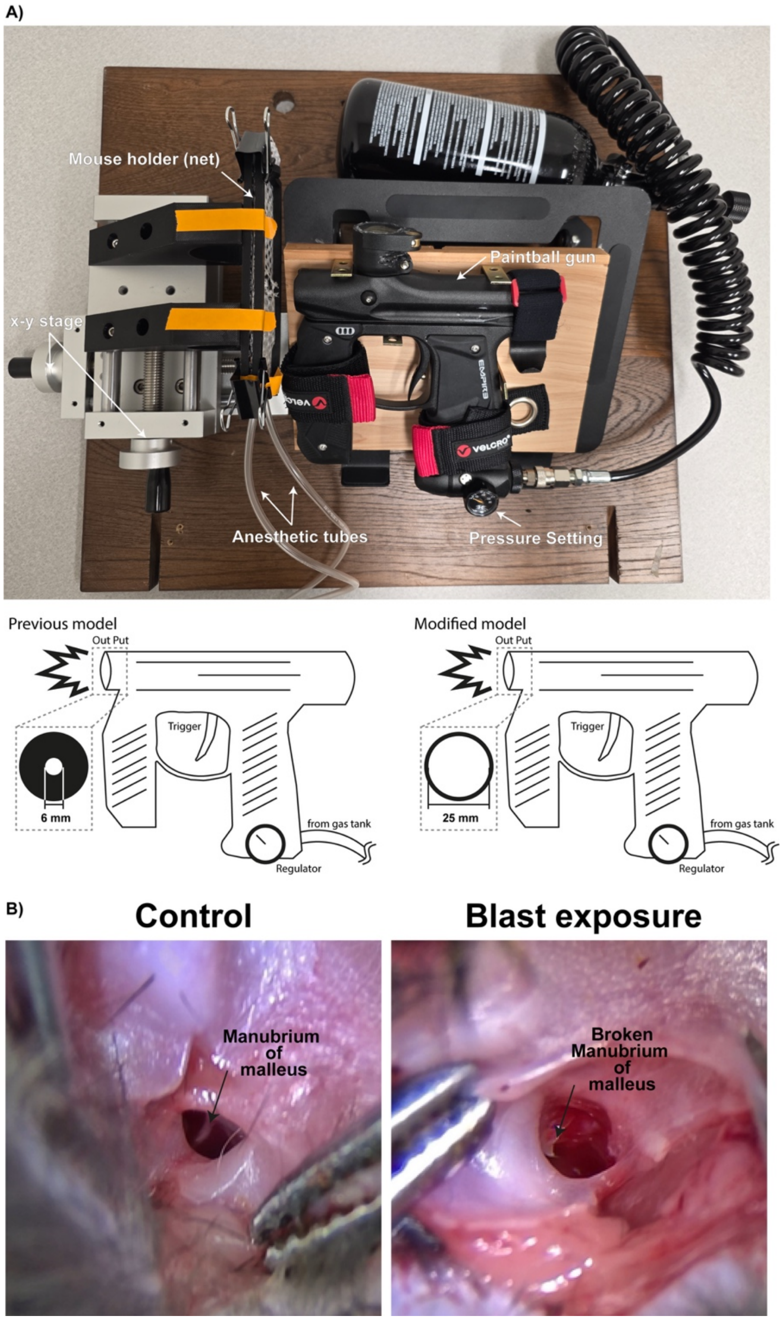

Supplementary figure 1. Modified blast gun model caused perforation of tympanic membrane. A) Modified Blast gun used in this research. A mouse holder with net was mounted on the x-y manipulator stage on the left of the photo and paint ball gun was mounted height adjustable stage on the right of the photo. The paint ball connected to compressed gas tank with gas tubing. The detail of modification from original device is illustrated at the bottom **B)** the perforation of tympanic membrane was checked right after blast exposure under stereomicroscope. While naïve control without any blast exposure showed intact tympanic membrane, it was perforated in the mice exposed to blast.

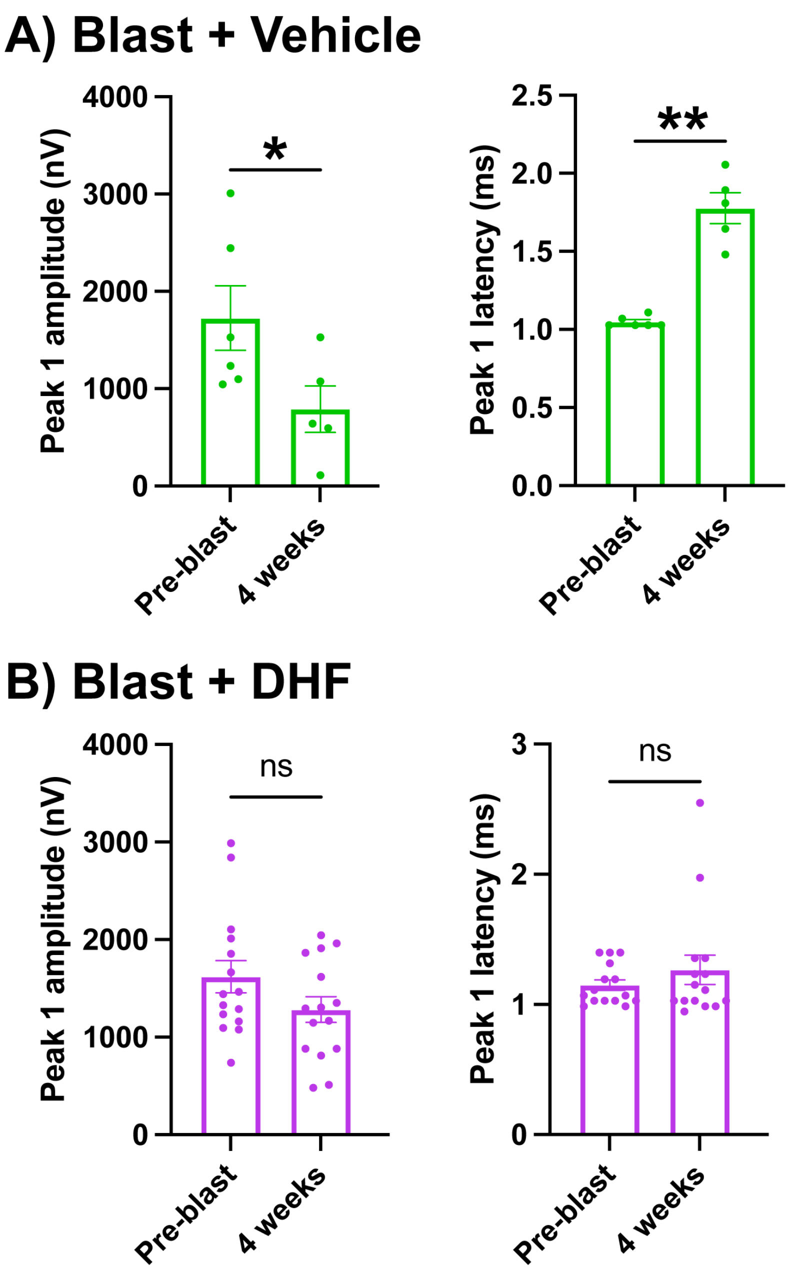

Supplementary figure 2. Recovery of ABR wave form at peak 1 after nanoparticle treatment. In addition to ABR threshold, ABR wave form at peak 1 was further validated. Consistent with ABR threshold alteration after blast exposure on control group, **A)** the peak 1 amplitudes were significantly reduced and latencies were significantly delayed after 1 month of recovery period. On the other hand, **B)** peak 1 amplitudes and latencies recovered to the normal level after 1 month of recovery period on DHF-nanoparticle treated group. To acquire statistical significancy, paired t-test was conducted and the significancy was presented as asterisk. *, $p<0.05$; **<0.01, ns, not significant.
